# Experimental Evaluation of Radar Sensor Mounting Height on Vehicle Active Safety Systems Performance

**DOI:** 10.3390/s26165060

**Published:** 2026-08-10

**Authors:** Michał Dusza, Łukasz Ugarenko, Peng Mei, Paweł Skruch

**Affiliations:** 1 Faculty of Electrical Engineering, Automatics, Computer Science and Biomedical Engineering, AGH University of Krakow, al. Mickiewicza 30, 30-059 Krakow, Poland; peng.mei@agh.edu.pl (P.M.); pawel.skruch@agh.edu.pl (P.S.); 2 Aptiv, 30-707 Krakow, Poland; lukasz.ugarenko@aptiv.com; 3 Politecnico di Milano, Piazza Leonardo da Vinci 32, 20133 Milan, Italy

**Keywords:** radar, radar detection, automotive, active safety

## Abstract

The paper investigates the impact of the height of the automotive radar mounting on the detection performance of vehicle active safety systems, with particular emphasis on multipath interference. Multipath occurs when radar signals are reflected by surfaces such as road pavement, leading to signal oscillations, false detections, or temporary loss of target tracking, which can degrade the reliability of active safety functions. The controlled experiments were conducted using mid- and long-range radar sensors mounted at heights ranging from 20 to 120 cm. Standardized targets, including pedestrian dummies, a Global Vehicle Target, and a corner reflector, were used. Straight-line approach scenarios at 30 km/h on a 250 m test track were evaluated using detection filtering and Radar Cross-Section analysis. The results demonstrate that low mounting heights (20–40 cm) significantly intensify multipath effects, whereas higher mounting positions (100–120 cm) reduce the detectability of low-profile obstacles. The best overall performance across the evaluated sensors in the controlled straight-line test scenario was achieved within the 40–100 cm mounting-height range, providing a good balance of detection reliability for both vehicles and pedestrians.

## 1. Introduction

Over the past few years, Advanced Driver Assistance Systems (ADAS) safety regulations, such as the GSR (Regulation (EU) 2019/2144), have mandated features that help prevent road accidents [1,2]. As a result, every new vehicle must be equipped with sensors such as cameras, radars, or both, depending on each Original Equipment Manufacturer’s (OEM’s) strategy and the desired ADAS system complexity [3,4,5]. Because vehicle designs and dimensions vary significantly, these sensors may be installed at different heights and positions. Consequently, even identical sensor types may deliver data from the surrounding area differently when installed higher or lower, due to the physics of electromagnetic wave propagation.

One of the key challenges in designing radar systems is the phenomenon of multipath—multiple reflections of electromagnetic waves from various surfaces—which can lead to false detections or a temporary loss of tracking. Mounting height is one of the critical factors that influence the severity of this phenomenon.

Modern vehicles exhibit a significant diversity in radar mounting heights. In sports cars, sensors are typically installed very low, close to the ground to fit within aerodynamic body designs. In contrast, pickup trucks and heavy-duty vehicles often position radar sensors much higher, sometimes more than one meter above ground, due to their larger front profiles and elevated chassis. Excess mounting height can cause the vehicle to fail to detect low obstacles close to the vehicle, such as small cars or elements of road infrastructure. In contrast, mounting the radar too low can lead to undesirable system behavior, such as false detections caused by road surface reflections or interference from terrain irregularities.

Prior research on automotive radar multipath has focused mainly on signal-level modeling, compensation of ground-reflection effects, and the influence of indirect propagation paths on target localization and direction-of-arrival estimation [6,7,8,9]. Other studies have shown that multipath can also be exploited for estimating target height or improving environmental interpretation [10]. However, these works primarily address multipath at the signal-processing or perception level. In contrast, the present study considers the problem from a system-integration perspective and investigates how radar mounting height itself changes the severity of multipath-related detection outages. In this sense, mounting height should be regarded not only as a packaging constraint, but also as a first-order design parameter that interacts with downstream mitigation methods.

To address this gap, a series of controlled test runs was conducted, recording radar data for various sensor mounting configurations and detection targets. The radar was mounted at heights ranging from 20 to 120 cm, while the detection targets included an adult pedestrian, a child pedestrian, a vehicle, and a corner reflector. The analysis compares the signal characteristics and detection performance as a function of mounting height.

The paper is organized as follows. The next section presents the characteristics of automotive radar sensors. This is followed by a description of the experimental setup, including the measurement and analysis methodology. The results obtained are presented and discussed in the following section. The paper is concluded in the final section.

## 2. Automotive Radar Sensors

Radar (*Radio Detection and Ranging*) is a sensing technology that transmits electromagnetic waves, which are reflected by surrounding objects and static structures in the environment. The returned electromagnetic echoes are captured by the radar antennas and processed by a digital signal processing (DSP) unit, where the signals are typically transformed from the time domain to the frequency domain. Based on transformed signals, radar signal processing algorithms extract relevant information from clutter and noise.

Several types of radar sensors exist; however, Frequency-Modulated Continuous-Wave radars (FMCW) are most commonly employed in the automotive industry. FMCW radar systems are capable of simultaneously measuring the radial distance to a target and its relative velocity with respect to the host vehicle [11,12,13]. In addition, FMCW automotive radars often employ multiple transmitting and receiving antennas. This configuration of the antenna array enables the estimation of the azimuth angle of the target in the horizontal plane [5,14].

Note that the range, relative velocity, and angle associated with a reflection point on an object jointly constitute a single radar detection. Consequently, an FMCW radar may generate multiple detections for a single physical target (e.g., a vehicle). A single scan of the radar field of view therefore results in a point cloud of detections. The number of detections is typically limited to 32 to 64 per scan.

### 2.1. Radar Cross-Section

Radar Cross-Section (RCS) is a fundamental parameter that describes how effectively an object reflects electromagnetic energy back toward the radar receiver. In essence, it represents the visibility of a target to the radar system. A higher RCS means that the object returns a stronger signal, making it easier to detect. It depends on geometry, material, angle of incidence, polarization, and frequency.

Typical RCS values vary widely [15,16]. Vehicles often exhibit RCS values of several square meters, but these values fluctuate with aspect angle due to complex geometries and distributed scattering centers [17,18,19]. Pedestrians have a much lower and more variable RCS, often in the range of −10 to −3 dBsm for adults and −13 to −7 dBsm for children at the radar frequency of 77 GHz. Standardized Euro NCAP targets replicate these characteristics to ensure repeatable and comparable testing conditions. The Global Vehicle Target (GVT) mimics a compact passenger car, while the corner reflector serves as a calibration reference with a stable RCS of approximately 10 dBsm, independent of orientation.

For an ideal trihedral corner reflector with an edge length *a* and a wavelength λ, the RCS σ can be expressed as follows:

(1) $$\sigma =\frac{4\pi a^{4}}{3\lambda^{2}}\;.$$

This predictable behavior makes corner reflectors invaluable for validating radar performance and analyzing multipath effects [20].

### 2.2. Multipath Propagation

**Figure 1:**
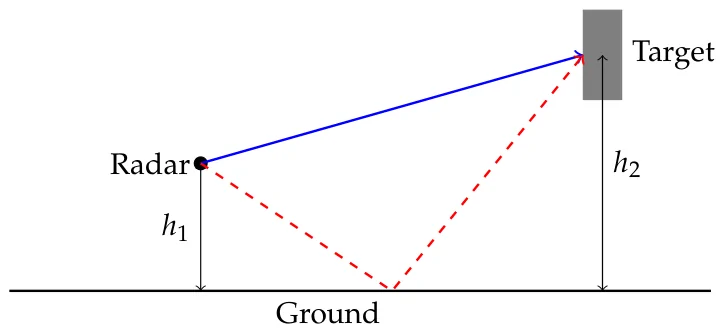
Illustration of direct and ground-reflected propagation paths contributing to multipath interference.

Multipath refers to the situation in which radar signals reach the receiver not only via the direct path from the target but also through one or more indirect paths caused by reflections from surfaces such as the road, infrastructure, or nearby objects (see Figure 1). These additional propagation paths introduce phase shifts, amplitude variations, and time delays, resulting in interference between direct and reflected signals [7,8,10,19].

When a radar transmits an electromagnetic wave toward a target, part of the energy travels directly to the object and back (direct path). However, other portions of the wave may reflect off the ground or other surfaces before reaching the target or returning to the radar [21,22]. Each reflected path has a different length, which means that the returned signals arrive with different phases. The superposition of these signals can lead to constructive interference (signal amplification) or destructive interference (signal attenuation), causing oscillations in the received signal strength.

In the paper [6], the author proposed a mathematical equation to illustrate the phenomenon of multipath propagation. If $P_{r}$ is the return power and $P_{f}$ is the transmitted power, a multipath phenomenon model can be described by the equation

(2) $$\frac{P_{r}}{P_{f}}=\frac{\sigma_{o}}{{\left(4\pi \right)}^{3}}\frac{G{\left(\eta_{1}\right)}^{2}\lambda^{2}}{\delta_{1}^{4}}{\left|1+R^{\prime }e^{-jk\Delta \delta }\right|}^{4}\;,$$

where $\sigma_{o}$ denotes RCS, $G(\eta_{1})$ is the antenna gain, λ indicates the wavelength, $\delta_{1}$ represents the distance to the target along the direct path, $R^{\prime }$ is the reflection coefficient of the additional path and Δδ is the distance difference between direct and indirect paths. The term ${\left|1+R^{\prime }e^{-jk\Delta \delta }\right|}^{4}$ captures the interference between the direct and reflected paths.

When the phase difference causes destructive interference, the received signal drops significantly, creating detection gaps. In contrast, constructive interference amplifies the signal, sometimes producing ghost targets. The height of the mounting directly changes the geometry that determines Δδ and the angular conditions that affect both antenna gain and the reflection of the ground. As a result, mounting height influences both the frequency of RCS oscillations over distance and the likelihood of detection gaps. An additional geometric effect appears in close range: if the radar is mounted far below or above the effective height of the target and the vertical field of view is limited, the radar beam may not fully illuminate the target at short distances. This can lead to reduced RCS and even missing detections in the near field, independent of classic interference, because the target is not adequately within the beam.

As illustrated in Figure 1, four distinct propagation paths between the radar and the target can be identified: (1) *Radar - Object - Radar* (direct path); (2) *Radar - Object - Ground - Radar*; (3) *Radar - Ground - Object - Radar*; (4) *Radar - Ground - Object - Ground - Radar*. According to the literature [6], under real sensor operating conditions, the RCS coefficient exhibits oscillatory behavior. This leads to large variations in the signal amplitude, which may result in periodic detection loss due to the insufficient signal power received by the radar.

## 3. Materials and Methods

### 3.1. Tested Radar Sensors

Two production automotive radar sensing units were evaluated: a medium-range radar (MRR) and a long-range radar (LRR). Table 1 reports only the principal parameters relevant to the interpretation of the results. Publicly available values are provided where available. Parameters that are not publicly disclosed for the tested configurations are marked accordingly. The study should therefore be interpreted as a black-box experimental case study of two production radar sensing units.

**Table 1:** Principal parameters of the tested radar configurations. Parameters not available in public documentation are marked as not publicly disclosed [23,24,25].

Parameter	MRR	LRR
Radar class	Medium-range automotive radar	Forward-facing long-range automotive radar
Operating frequency	76.005–76.915 GHz	Not publicly disclosed
Bandwidth	Not publicly disclosed for the tested configuration	Not publicly disclosed for the tested configuration
Horizontal coverage used in Figure 2	Approximately $\pm 45^{\circ }$	Approximately $\pm 45^{\circ }$
Elevation coverage	Approximately $\pm 5^{\circ }$	Approximately $\pm 10^{\circ }$
Angular resolution	Approximately $1^{\circ }$	Approximately $1^{\circ }$
Update rate	Not publicly disclosed	Not publicly disclosed
Tx/Rx or MIMO configuration	Not publicly disclosed	Not publicly disclosed
Range-rate resolution	0.3 m/s	Not publicly disclosed
Output used in this study	Untracked sensor-level detections	Untracked sensor-level detections

**Figure 2:**
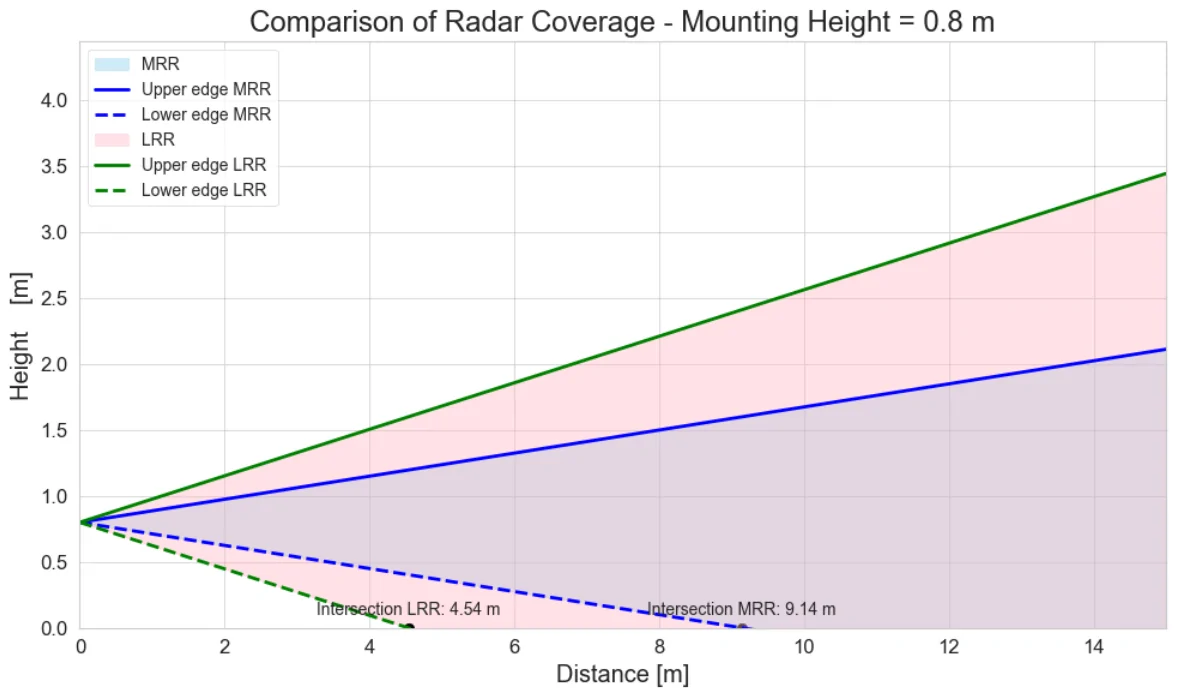
Nominal effective coverage characteristics of the MRR and LRR sensors used in the tests. The shown boundaries represent the main operational coverage used for interpreting the measurements; side-lobe regions are not shown.

### 3.2. Measurement Scenario and Procedure

The experiments were conducted on a dedicated test track designed for repeatable ADAS scenarios. All tests were performed under controlled conditions on a dry road surface, with no additional objects present in the measurement area. For each run, the target was positioned on the centerline of the straight-line approach scenario, and the radar orientation was kept constant except for the adjusted mounting height. The driving scenario consisted of a straight-line approach toward a stationary target at a constant speed of 30 km/h. Data collection began 120 m from the target and continued until the target position was reached, with the straight trajectory chosen to minimize environmental variability and simplify interpretation of range-dependent behavior. The length of the track exceeded the required 120 m, allowing data collection to start once the vehicle stabilized at the target speed, which improves repeatability.

### 3.3. Target Types

**Figure 3:**
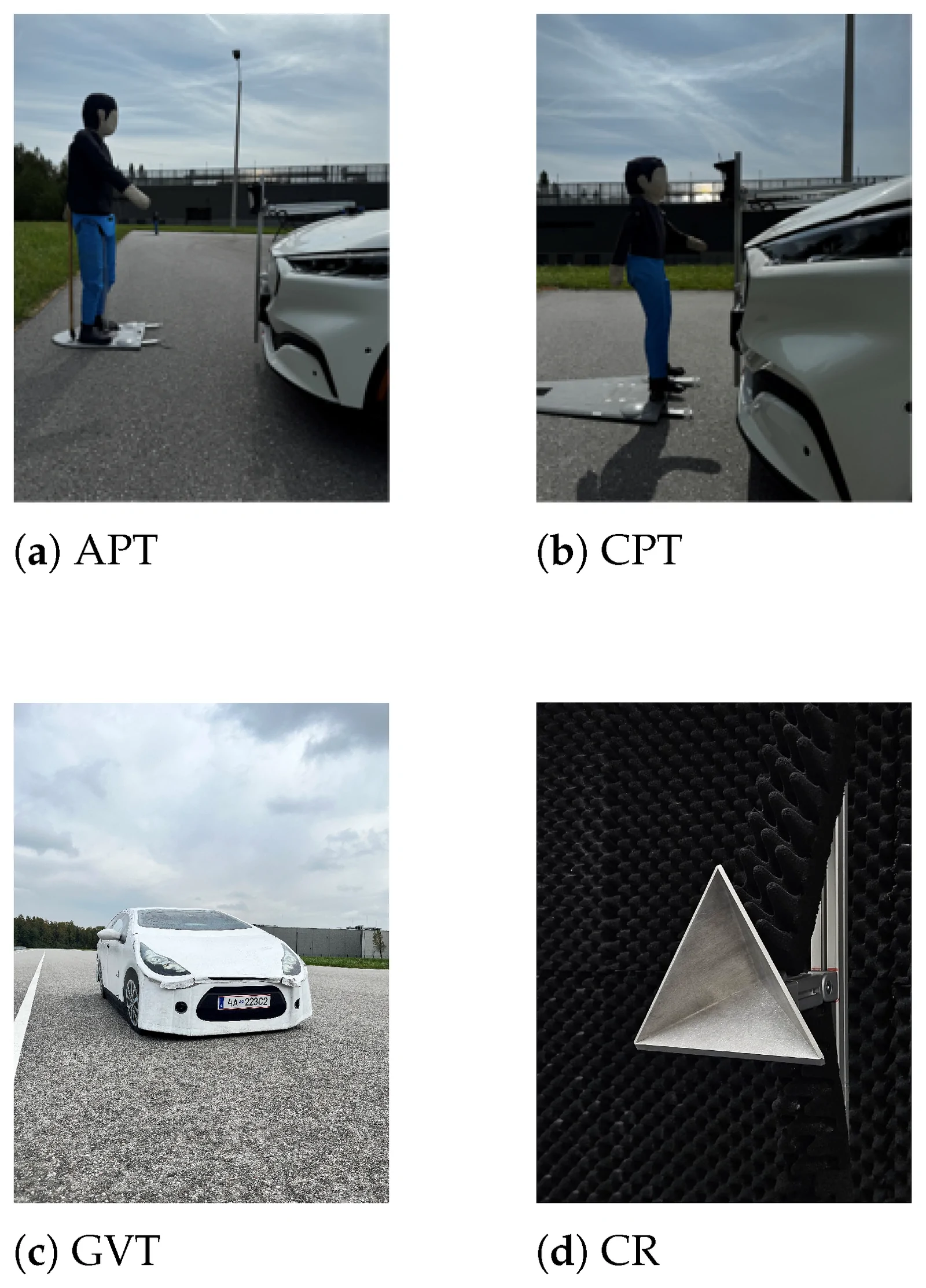
Target types used in the experimental campaign: adult pedestrian target (APT), child pedestrian target (CPT), Global Vehicle Target (GVT), and trihedral corner reflector (CR).

Four targets were used during the experiments (see Figure 3). There were three commonly used standardized objects in the Euro NCAP testing: an adult pedestrian target (APT), a child pedestrian target (CPT), and a global vehicle target (GVT). The APT represents a pedestrian of 1.75 m tall with an RCS, at a work frequency of 77 GHz, typically around −6 dBsm on average, varying approximately from −10 to −3 dBsm depending on pose and observation angle [26]. The CPT represents a smaller child-like silhouette around 1.1 m tall with lower reflectivity, with RCS values in the range of approximately −13 to −7 dBsm, reflecting the increased difficulty in detecting vulnerable small road users [26]. The GVT is a deformable standardized vehicle surrogate designed to mimic the reflective properties of a typical hatchback at viewing angles while remaining safe for testing. Its materials and structure emulate elements of real vehicles, such as lamps, panels, and tires, in both the radar and the infrared response [27]. The fourth target was a corner reflector with a nominal RCS of 10 dBsm, specifically included as a reference with stable reflectivity to diagnose multipath behavior.

### 3.4. Adjustable Radar Mounting Setup

**Figure 4:**
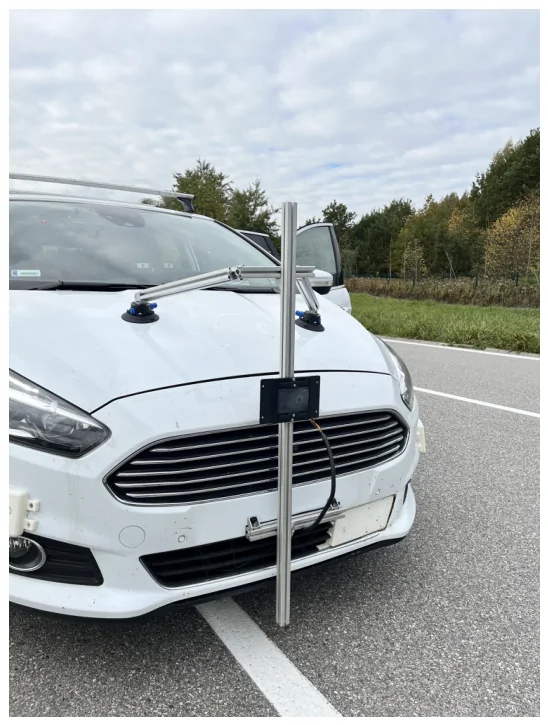
Adjustable radar mounting bracket used to vary the sensor installation height.

To study the mounting height, a dedicated adjustable mounting rig was constructed (see Figure 4) to allow rapid and repeatable changes in the radar position. The height was varied from 0.20 to 1.20 m in increments of 0.10 m, spanning typical mounting positions from low sports-car installations to high commercial vehicle placements. The rig also allowed for orientation corrections in roll, pitch, and yaw with an adjustment capability of approximately $\pm 1^{\circ }$, helping to ensure that the results reflect height effects rather than misalignment.

After each height change, the radar orientation was rechecked and adjusted using the mounting rig to keep the sensor boresight aligned with the straight-line approach axis. The mounting height was verified with a measuring tape, while the sensor alignment and level were checked using a spirit level. Yaw and boresight alignment were checked geometrically with respect to the straight-line approach axis: the target was positioned on the road centerline, and the vehicle was driven along this line directly toward the target. This procedure ensured that the radar boresight remained aligned with the nominal target approach direction for all tested mounting heights. The stated $\left(\pm 1^{\circ }\right)$ value should be interpreted as an estimated alignment uncertainty after manual adjustment rather than as a directly measured angular error. For each mounting height, two runs were conducted to verify the repeatability of the tests and the observed phenomena.

### 3.5. Evaluation Metrics

The analysis was performed using untracked sensor-level detections. Tracked-object outputs from the radar tracking layer were not used. This choice was made to evaluate detection continuity directly at the detection level and to avoid masking short detection outages by tracker prediction or smoothing. For each scan, target-associated detections were selected based on consistency with the expected target position in the straight-line approach scenario.

Two main outcome categories were considered: RCS stability and detection continuity. The stability of the RCS was assessed by inspecting the RCS as a function of the range for each height and by characterizing the distribution of the RCS values using kernel density estimation. This distribution-level view is useful because multipath can create multi-modal behavior, with the signal repeatedly switching between higher and lower power regimes.

To evaluate detection continuity, detections were first associated with the expected target position based on the straight-line scenario and filtered using spatial consistency in range and azimuth relative to the target lane. The continuity analysis was then performed scan by scan. A detection gap was defined as a sequence of consecutive radar scans in which no target-associated detection was present, bounded by scans in which the target was detected. For each gap, its length was expressed as the covered range interval between the last scan with a valid target-associated detection before the gap and the first scan with a valid target-associated detection after the gap. Based on these gaps, three quantities were extracted for each run: the number of gaps, the maximum gap length, and the cumulative gap length.

### 3.6. Statistical Treatment and Repeatability

For each radar mounting height and target configuration, two independent runs were performed under identical experimental conditions. The maximum detection gap, the number of gaps, and the cumulative gap length were calculated separately for each run, and the final reported values correspond to the mean of the two runs. The same qualitative trends were observed in both repetitions, which supports the repeatability of the reported height-dependent effects. Given that only two runs were available for each configuration, the analysis is descriptive and does not aim at statistical inference. Data processing and visualization were performed in Python 3.9.11 using NumPy, pandas, SciPy, matplotlib, and seaborn.

## 4. Results and Discussion

### 4.1. RCS Dependence on Range and Height

**Figure 5:**
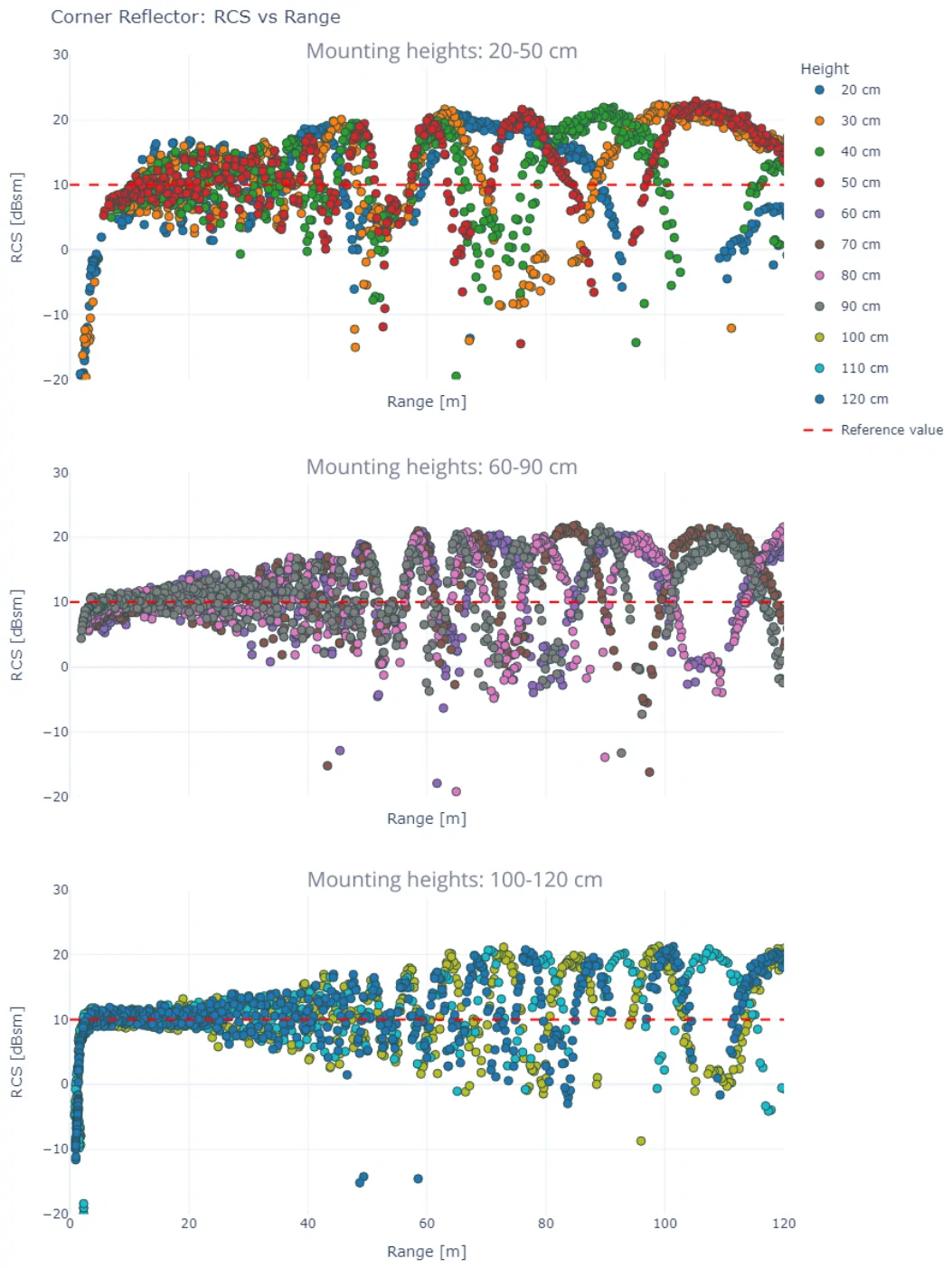
Measured RCS values of the corner reflector as a function of distance for selected LRR mounting heights.

Across targets and sensors, the experiments show a consistent qualitative signature of multipath: the measured RCS oscillates with range, and the character of the oscillations changes with distance (see Figure 5). As the range increases, the oscillation frequency decreases, meaning that the signal is more slowly transitioning between constructive and destructive interference. At the same time, the oscillation amplitude tends to increase with distance, producing deeper minima at longer ranges. This combination is unfavorable for the continuity of detection because it increases the chance that the signal remains below the detection threshold for a longer range interval at far distances.

The mounting height modifies these signatures, which can be observed directly in Figure 5. Low mounting positions tend to produce slower oscillations and larger or more frequent deep minima, while higher mounting positions generally increase the oscillation frequency, reducing the range of destructive regions. Additionally, the experiments reveal a near-field effect at low mounting heights: when radar height is far below the effective target height, the elevation field of view can become too narrow to fully illuminate the target at very short distances. In that regime, the radar may detect only part of the object, which can reduce the RCS and even cause near-field loss of detections. The same qualitative dependence on mounting height was observed in both runs for each evaluated configuration.

Figure 5 illustrates the experimentally observed RCS oscillations as a function of range for different LRR mounting heights. The oscillatory behavior is interpreted qualitatively using the multipath model from Equation (2), where constructive and destructive interference depend on the path-length difference between the direct and ground-reflected propagation paths. A quantitative overlay of theoretical dependencies was not added because it would require additional parameters that were not independently measured. Therefore, Figure 5 is used as an experimental illustration of the multipath behavior rather than as a direct validation of a fully parameterized theoretical model.

### 4.2. Corner Reflector

The corner reflector provides the clearest diagnostic case because it has a high and comparatively stable nominal reflectivity and does not introduce the complex scattering variability associated with pedestrian or vehicle targets. At low mounting heights of 20–40 cm, the reflector exhibits the longest detection outages. These outages occur in range regions where deep RCS minima are observed, supporting the interpretation that destructive ground-reflection interference can directly translate into missed detections even for a high-RCS target.

As the mounting height increases beyond approximately 60 cm, the gap length decreases substantially. The most stable configuration is obtained when the radar is mounted close to the reflector height of about 115 cm. This behavior is consistent with improved vertical alignment between the radar coverage and the dominant scattering region of the reflector, together with a reduced influence of destructive multipath fading within the evaluated range interval.

The reduction in measured RCS at very short distances should not be attributed only to insufficient target illumination. A trihedral corner reflector reaches its nominal RCS only in the far-field region. The Fraunhofer distance can be approximated as

(3) $$R_{F}\approx \frac{2D^{2}}{\lambda }\;,$$

where *D* is the characteristic reflector dimension and λ is the radar wavelength. For a corner reflector with an edge length of approximately 0.10 m operating near 77 GHz, the corresponding far-field distance is approximately 5 m. Therefore, at shorter ranges, the effective RCS may be lower than the nominal value even for a geometrically well-aligned reflector. This near-field effect acts together with partial illumination, vertical field-of-view limitations, and multipath-induced destructive interference.

### 4.3. Adult Pedestrian and Child Pedestrian Targets

**Figure 6:**
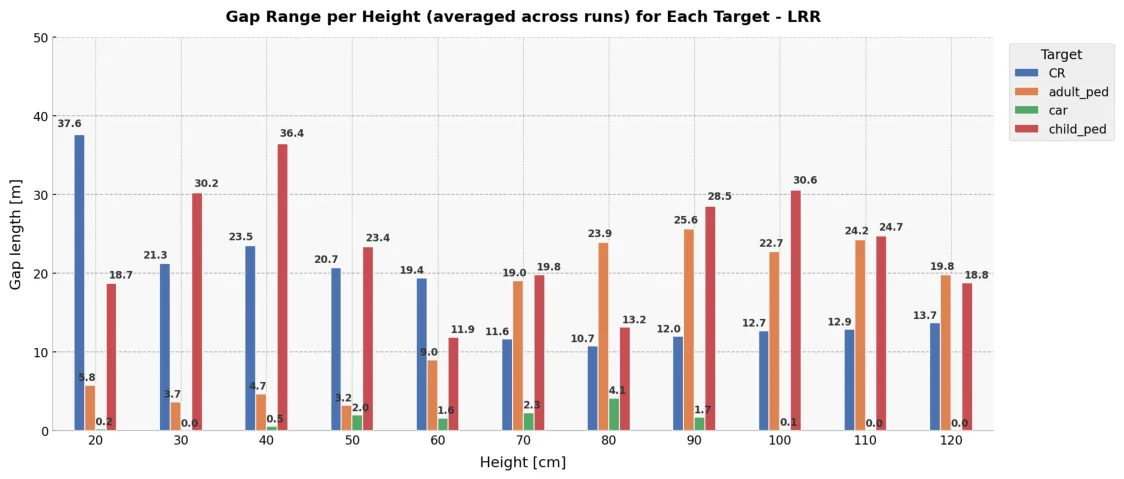
Detection gaps for all evaluated targets measured with the LRR across radar mounting heights.

**Figure 7:**
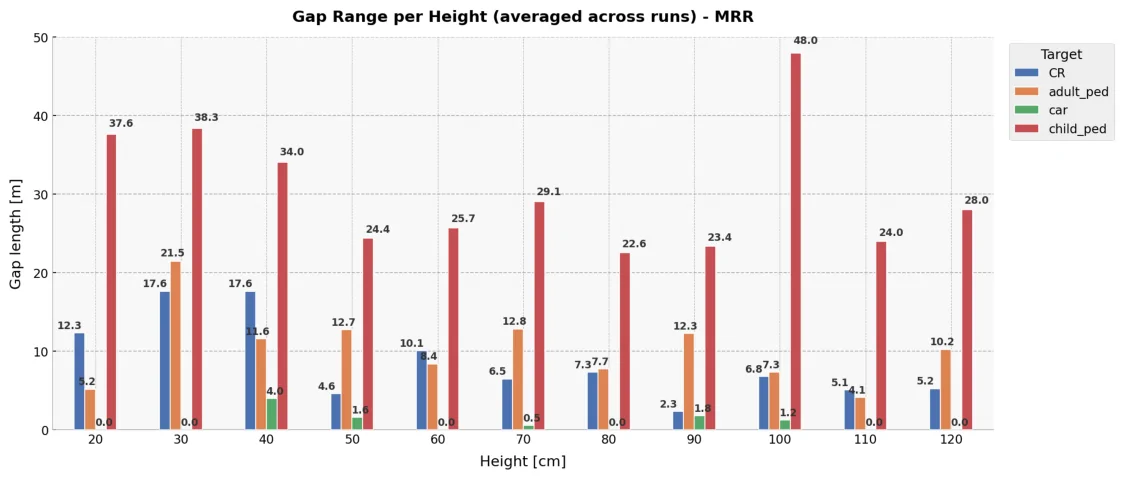
Detection gaps for all evaluated targets measured with the MRR across radar mounting heights.

Pedestrian targets, both adult and child, represent the most challenging objects for radar detection. The child pedestrian exhibits relatively uniform detection gaps throughout the mounting height range (Figure 6 and Figure 7).

The pedestrian results reveal that the influence of mounting height is strongly target-dependent. This behavior is expected because the adult pedestrian target and the child pedestrian target differ not only in total RCS, but also in the vertical distribution of scattering centers. The child pedestrian target has a lower height and lower RCS, which makes it more sensitive to vertical field-of-view limitations and local destructive interference. In contrast, the adult pedestrian target presents a taller scattering structure, so different parts of the body can dominate the radar response at different mounting heights and ranges. For the LRR, the child pedestrian target shows comparatively favorable continuity at lower mounting heights, around 20–40 cm, whereas the adult pedestrian target performs more favorably around 60–80 cm. This suggests that the optimum mounting height depends on the alignment between the sensor elevation coverage and the dominant scattering regions of the target. For the MRR, the same target-dependent behavior is visible but appears more irregular, which can be attributed to the combined effects of narrower elevation coverage, target RCS variability, and the actual antenna beam pattern. In particular, local anomalies, such as the child pedestrian result around 100 cm mounting height, may be influenced by side-lobe reception or by a non-ideal alignment between the target scattering centers and the main beam. Therefore, these local extrema should be interpreted cautiously and should not be used as stand-alone design optima.

### 4.4. Global Vehicle Target

The Global Vehicle Target is the least sensitive object in the study. The maximum gap length remains small throughout the full mounting height range. The maximum gaps do not exceed approximately 5 m. This robustness is consistent with the multi-scatter nature of vehicle-like targets, where multiple reflective components provide redundancy. Even if multipath causes fading for one scattering path, other components can remain detectable, preventing extended loss of detections.

### 4.5. Comparison of LRR and MRR

**Figure 8:**
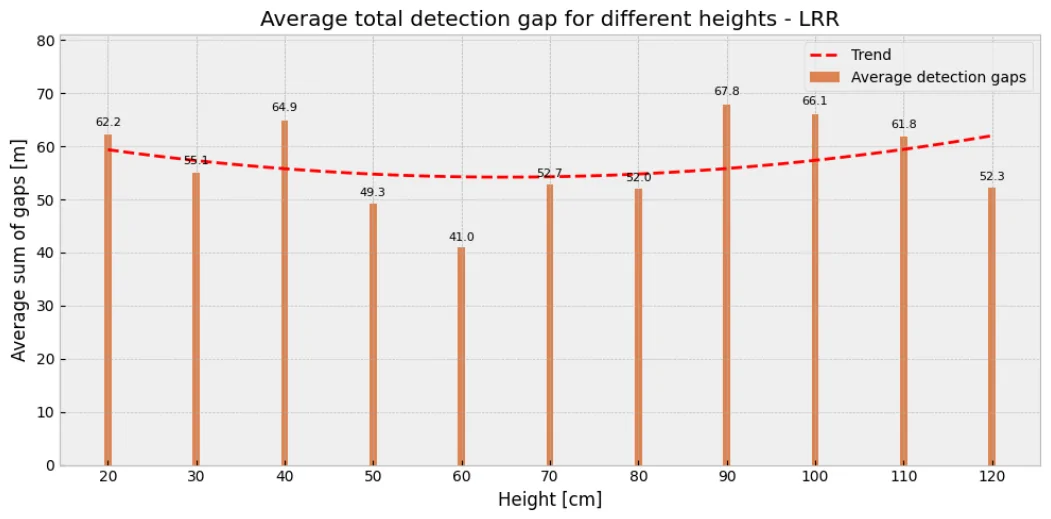
Average cumulative detection gaps for selected target classes measured with the LRR.

**Figure 9:**
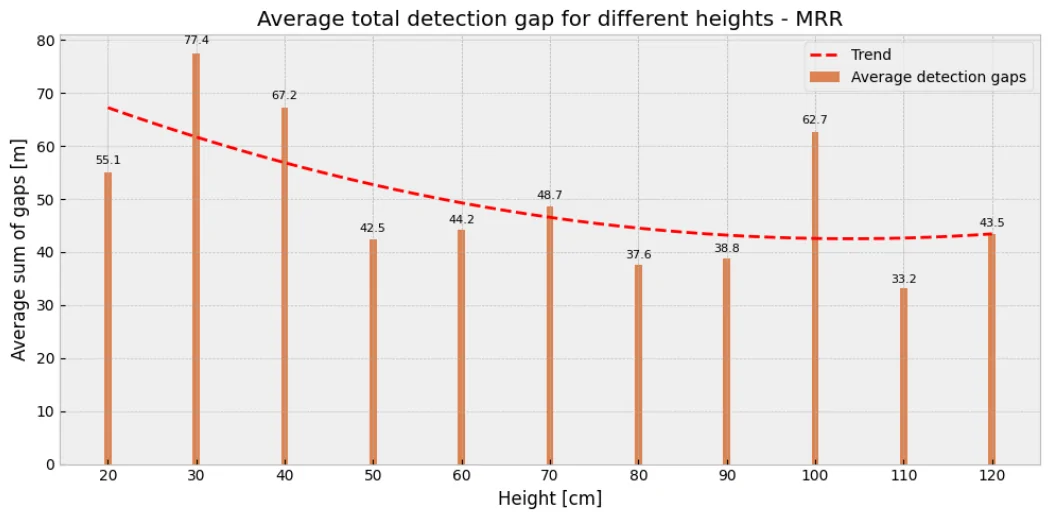
Average cumulative detection gaps for selected target classes measured with the MRR.

Comparing the two sensors reveals that the qualitative influence of height is consistent. Figure 8 and Figure 9 present the average cumulative detection gaps for three targets: GVT, APT and CPT. The corner reflector was omitted, as a perfectly reflective object does not occur under real-world road conditions. For the LRR, a mild U-shaped trend is noticeable, indicating that mounting heights in the mid-range provide the most favorable performance. In contrast, for the MRR, the trend is more pronounced, with a clear reduction in detection gaps when the sensor is mounted at approximately 40 cm (excluding the outlier observed at 100 cm).

The similarity of the RCS oscillation structure across sensors indicates that the dominant driver is the radar-target-ground geometry rather than a sensor-specific artifact. Differences in elevation coverage remain relevant in the near field, where a broader vertical beam coverage can reduce the risk of an object leaving the elevation field of view at small ranges; however, it does not eliminate ground multipath interference.

Figure 8 and Figure 9 should be interpreted as system-level summaries rather than as a complete basis for selecting the radar mounting height. Averaging over target classes is useful for identifying global trends, but it can hide safety-critical target-specific behavior. In particular, pedestrian targets may be affected by mounting height differently than the Global Vehicle Target because of their lower RCS and different vertical scattering structure. For this reason, the averaged gap values should be considered together with the target-specific results shown in Figure 6 and Figure 7. The comparison also shows that the mounting-height effect cannot be explained by multipath geometry alone. The path-length difference between the direct and ground-reflected components determines where constructive and destructive interference can occur, but the severity of detection gaps is also shaped by sensor-specific parameters such as antenna beamwidth, elevation coverage, angular resolution, transmit power, bandwidth, and internal detection thresholds.

## 5. Conclusions

This paper investigates how the height of an automotive radar mounting influences multipath-related RCS modulation and detection continuity in a controlled straight-line approach scenario. The experiments show that the measured RCS can be strongly oscillatory versus range and that mounting height shapes the oscillation structure through the radar-target-ground geometry.

Low mounting heights tend to produce the largest and most persistent destructive-interference regions and are associated with substantial detection gaps. The corner reflector results provide a clear reference: at mounting heights of 0.20 to 0.40 m, maximum gaps reach about 26 m, while mounting near the reflector height of around 1.15 m yields the most stable performance, with maximum gaps below 6 m and only a few gaps in total.

For pedestrian targets, the impact of mounting height differs between radar types and target classes. In the case of the LRR, the poorest detection continuity is observed for mounting heights above 0.60 m. In contrast, for the MRR, the worst performance occurs at lower mounting heights up to 0.50 m, while the distribution of detection losses remains relatively uniform across the evaluated heights. The child pedestrian target, characterized by lower RCS and lower dominant scattering regions, shows higher overall variability and local worst-case heights. In contrast, the global vehicle target remains robust across heights, with maximum gaps below 5 m and only a few interruptions, which is consistent with the presence of multiple reflective components.

The study indicates that no single mounting height is universally optimal for all target classes or radar configurations. Although increased height often reduces the severity of outages under multipath conditions, excessively high placement can limit near-field coverage for low obstacles due to elevation field-of-view constraints. Mounting height should therefore be treated as a system-level integration parameter whose effect depends on the radar type, antenna coverage, target height, target RCS, and the distribution of scattering centers. Mid-range mounting positions provide a favorable compromise in the tested straight-line scenario, but the optimum may shift for different radar hardware, bumper geometries, road surfaces, target classes, or signal-processing configurations.

Publicly available standardization reports and component-level radar documentation typically describe frequency bands, representative radar parameters, and general system capabilities, but they do not provide a universal mounting-height recommendation applicable to all vehicle platforms. Public target-validation procedures, such as Euro NCAP GVT radar-reflectivity measurements, define reference sensor positions and alignment tolerances for specific measurement setups; however, these values should not be interpreted as general design recommendations for all production vehicles. The present work therefore complements existing public information by experimentally quantifying how mounting height affects detection continuity and RCS stability under controlled conditions.

This study is limited to a controlled straight-line approach scenario designed to isolate the influence of mounting height on radar multipath behavior. As a result, the conclusions should not be directly generalized to all driving situations. In more complex scenarios, such as curved motion, dense traffic, or varying road and weather conditions, the radar-target-ground geometry may differ substantially and alter the observed detection-gap patterns. Therefore, the identified favorable mounting ranges should be interpreted as design guidelines for controlled conditions rather than universal optima. Future work will extend the analysis to more realistic driving scenarios and additional environmental conditions.

## Data Availability

The original contributions presented in this study are included in the article. Further inquiries can be directed to the corresponding author.

## Abbreviations

The following abbreviations are used in this manuscript:

ADAS	Advanced Driver Assistance Systems
APT	Adult Pedestrian Target
CPT	Child Pedestrian Target
DSP	Digital Signal Processing
FMCW	Frequency-Modulated Continuous-Wave
GSR	General Safety Regulation
GVT	Global Vehicle Target
LRR	Long-Range Radar
MRR	Medium-Range Radar
OEM	Original Equipment Manufacturer
RCS	Radar Cross-Section
